# Top-down pulses reduce prey population sizes and persistence

**DOI:** 10.1038/s41598-018-27661-1

**Published:** 2018-06-19

**Authors:** Elizabeth A. Hamman, Michael W. McCoy

**Affiliations:** Department of Biology, East Carolina University, Greenville, USA

## Abstract

Resource pulses are well documented and have important consequences for population dynamics relative to continuous inputs. However, pulses of top-down factors (e.g. predation) are less explored and appreciated in the ecological literature. Here, we use a simple differential equation population model to show how pulsed removals of individuals from a population alter population size relative to continuous dynamics. Pulsed removals result in lower equilibrium population sizes relative to continuous removals, and the differences are greatest at low population growth rates, high removal rates, and with large, infrequent pulses. Furthermore, the timing of the removal pulses (either stochastic or cyclic) affects population size. For example, cyclic removals are less likely than stochastic removals to result in population eradication, but when eradication occurs, the time until eradication is shorter for cyclic than with stochastic removals.

## Introduction

Temporal variability in both-bottom up (e.g. masting trees or nutrient runoff following rare heavy rainfall, reviewed in^1,2^) and-top down (e.g. mobile predators that move among patches^3,4^ or consumer aggregations^5^) processes is likely the norm for most ecological systems, yet the consequences of top-down pulses on ecological dynamics are rarely explored (but see^6–8^). Explicitly incorporating temporal variation of top-down forces into theoretical models of population dynamics will add realism and improve our basic understanding of population dynamics and improve our ability to accurately predict ecological dynamics and equilibrium abundances in natural systems^9^. For instance, theoretical work on pulsed vaccination strategies demonstrates how pulsed, rather than continuous removals of susceptible individuals from a population can decrease disease persistence in SIR^10^ and age-structured transmission^11^ models. Studies of fisheries stock assessment models have shown that pulsed harvesting strategies produce smaller yields than continuous harvesting^12^. However, the effects of different frequencies of episodic predation or harvesting on the population dynamics and equilibrium abundances of populations have not been well explored.

Here, we incorporate temporal variation in consumer pressure by comparing pulsed and continuous removals on equilibrium population size and illustrate the potential implications of pulsed removals with an example application for managing an invasive species.

## Model and Analysis

To explore the effects of pulsed removals on prey population dynamics, we expand on the Schaefer harvest model^13^, which is a basic ordinary differential equation (ode) population model (Eq. 1) that assumes logistic population growth, where the rate of change in population *N* (*dN/dt*) is

1 $$\frac{dN}{dt}=rN(1-\frac{N}{K}),$$

with *r* as the intrinsic population growth rate and *K* the carrying capacity. We then remove, or harvest individuals from the population at rate *H*, both continuously (Eq. 2), and discretely (Eq. 3). In the case of the discrete removals, *δ* is a Dirac-delta function with magnitude *H* that occurs at intervals of γ.

2 $$\frac{dN}{dt}=rN(1-\frac{N}{K})-H$$

3 $$\frac{dN}{dt}=rN(1-\frac{N}{K})-\sum _{i=0}^{\infty }H{\delta }_{\gamma i}(t)$$

We compared these three population models using simulations where the same number of individuals were removed from a population and subsequent effects on the mean equilibrium population size was recorded. Thus, more frequent pulses were smaller (and more similar to continuous removal) than larger, less frequent pulses. The interval between each pulse was spaced either regularly (cyclic pulses) or stochastically (see Methods for general description and Supplementary Methods for R code).

**Figure 1:**
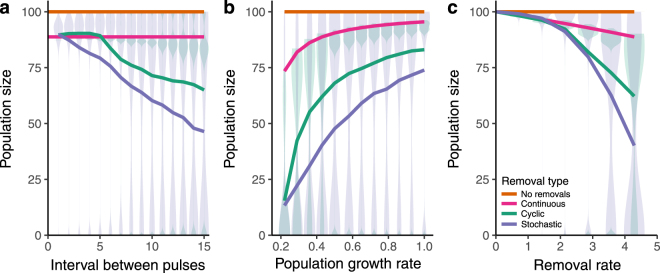
Effect of removals on equilibrium population size. Solid lines indicate mean population size, and shaded areas the spread of simulation values for pulsed removals. When pulses are frequent, there is little difference between cyclic removals (green lines), stochastic removals (purple lines), and continuous removals (pink lines) (Panel a). However, at small population growth rates (Panel b) and large removal rates (Panel c), pulsed removals lower equilibrium population size, greater than both cyclic and continuous removals. Unless varied, *H* = 4.3, *r* = 0.43, and *γ* = 12.9.

When pulses are frequent (and the number removed at each time step is small), there is little difference between the equilibrium population size of continuous and pulsed removals (Fig. 1a). However, as pulses become larger and less frequent, the population experiences a larger difference in average population growth rate due to the nonlinearity in the prey growth equation, resulting in lower equilibrium population sizes (see Supplementary Fig. S1). Less frequent pulses produce the greatest differences between continuous and pulsed removals when population growth rates are low (Fig. 1b) or removal rates are large (Fig. 1c). However, this relationship is governed by the timing of pulses. For example, at *H* = 4.29 and *r* = 0.43, continuous removals reduce the population size by an average of 11%, cyclic removals every 2.3 time steps reduce the population size by an average of 38%, and stochastic removals by an average of 60%. The disparity between cyclic and stochastic removals occurs because, on average, the cyclic removals allow for populations to recover before the next removal event (Supplementary Fig. S2). In addition to differences in mean population size, there are also differences in the distribution of equilibrium population sizes among the pulsed treatments. The equilibrium size of a population undergoing pulsed removals is often bimodal, with stochastic removal pulses exhibiting a larger range of population sizes and a larger amount of lower population sizes than cyclic removal pulses (Fig. 1). Overall stochastically timed removals were also more variable than cyclic removals.

In light of the bimodality of population sizes in pulsed removals, and in particular the high number of equilibrium population sizes at 0 (i.e. extinction or eradication), we further explored the implication of our models by simulating an invasive species management scenario. Semi-discrete models have been used to incorporate temporal variability in removal of invasive species and they can offer a better fit than continuous models^14^. Moreover, many removal strategies consist of pulses due to concentrated intense removal efforts^15^ and repeated volunteer days^16^. However, because pulsed removal strategies can reduce population equilibrium abundances (Fig. 1), the pulses themselves may be leveraged into an effective management strategy. Using the model described in Equations 1–3 we test the effect of pulsed removals on the likelihood of eradicating populations and on the time until eradication (when eradication occurs). While we did not explicitly model continuous removals, cyclic removals over short time intervals are very similar to continuous dynamics (compare pink and green lines at low intervals between pulses in Fig. 1a) as expected, and therefore in eradication simulations, cyclic pulses that are temporally close together (pink dashed lines) approximate continuous removal dynamics.

**Figure 2:**
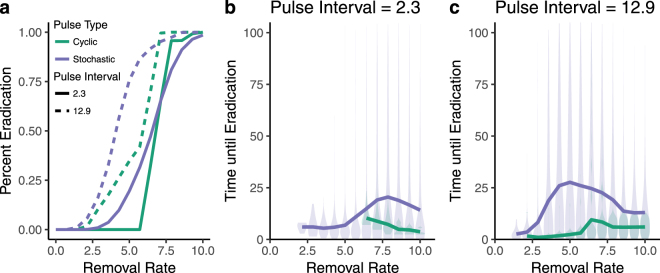
Effect of pulsed removals on invasive species management. The percent of simulations where eradication occurred (panel a) increased as removal rate increased for both stochastic (purple lines) and cyclic (green lines) timing of pulses. The time to eradication varied according to the timing of pulses and interval between pulses (panels b and c). Solid lines indicate mean population size, and shaded areas the spread of simulation values for pulsed removals. Eradications were more likely and occurred more quickly with larger, less frequent pulses (solid lines) than more frequent, smaller pulses (dashed lines), which approximate a continuous dynamic. In these simulations, *r* = 0.43.

Both the likelihood of eradication (percent of simulations where the species was eradicated, Fig. 2a), and the time to eradication (Fig. 2b,c) depended on the timing of pulses. Pulses that were infrequent (large interval between pulses) were more likely to result in eradication, particularly for stochastic pulses (Fig. 2a). Cyclic removals were sharply demarcated between removal rates that either never or always led to eradication. In contrast, stochastic pulses gradually increased the likelihood of eradication as removal rate increased. Moreover, the switch between successful and unsuccessful eradication occurred more quickly for larger, less frequent pulses, and cyclically timed pulses led to eradication more quickly than stochastic pulses (Fig. 2b,c). These patterns emerge because stochastic pulses that occur in rapid succession have a larger effect (even accounting for occasional longer recovery times) than episodic pulses that typically allow more recovery (see Supplementary Fig. S2).

## Discussion

While the effects and interactions of top-down and bottom-up factors have been frequently explored in the ecological literature, the effects of temporal variability have only been well explored from the bottom-up^2,17^. Just as resource pulses affect community dynamics and trophic interactions via changes to growth and reproduction rates^18^, prey switches^19^, and coexistence among competitors^20^, it is likely that variation in prey abundance that results from pulses of predation (via removals of prey) could also have important effects on food web interactions^21,22^. Here, we demonstrated how pulsed removals of individuals from a population undergoing logistic growth results in lower equilibrium population sizes than continuous removals (Fig. 1). Although the models explored in this study do not investigate effects on food web dynamics, our findings demonstrate how incorporating variation in the magnitude of top down processes can have important implications for prey populations, which likely cascade through food webs to affect community dynamics^7,23^. Future work should explore these effects, as well as how the effects of top-down pulses compare to pulses from the bottom-up.

One key assumption of our model was that removals of individuals from a population are constant and independent of population size. While this makes comparisons between models simple as a fixed number of prey are removed, and only timing differs, this is unrealistic for predators and other consumers that remove individuals at rates that scale with population size^24,25^. If the removals of individuals decrease in magnitude as population size decreases, the effect of removal pulses (or pulses in the rate of removal) will also decrease. This will reduce the negative effect of pulses on population sizes relative to continuous removals and lowers the likelihood of eradication. A second key assumption of our model is the assumption of logistic population growth. This nonlinearity in population growth causes removal pulses to lower equilibrium population sizes (Supplementary Fig. 1) due to reduced rates of population growth when recovering from pulsed removal events. However, when population growth is approximately linear, the changes in population growth rate over the pulse interval will be small, and the effect of relatively large and rare pulses will mimic the effects of small frequent pulses because both result in relatively small changes in population growth rate during recovery periods. While logistic growth is a reasonable assumption for most self-regulating populations, additional forms of both population growth and the relationship between removals and population size are interesting avenues for additional research.

Removal pulses also likely have important applications to management and conservation. For example, other studies of invasive species management have considered timing of management interventions with regards to life history stages^26^, compensatory growth^27^, or “press” vs., “pulse” removals. These studies show that while “press” (i.e. continuous) removals are typically more effective, they also require more effort^28^. However, we show that the effectiveness of continuous (i.e. press) and pulse removals, given fixed effort, varies depending upon magnitude of the removal strategy and the focal populations growth rate. For fixed effort, simulations with removals that are pulsed outperform continuous removals for both the likelihood of eradication and the time to eradication. Our study highlights that a better appreciation of the effects of temporal pulses of top-down effects in a theoretical framework can improve not only our understanding of basic population dynamics^23^, but may also have important implications for bio-control^29^, and invasive species and resource management.

## Methods

To test the effect of pulses on population size, we simulated Equations 1–3 in R. For Equations 1 and 2 (ODEs with no harvest and continuous harvest), we used the deSolve package, and for Equation 3, we simulated the solution of the ODE without removals (logistic population growth) between removal events. The intervals between removal events (*γ*) were timed either regularly (a removal event occurred at a given frequency), or stochastically, where the exact time between removal events was an exponential random variable with the mean time equal to that of the regularly timed removals. We simulated 100 timesteps, and while the time to equilibrium varied based on parameters, it generally occurred relatively quickly in the simulation (see Supplementary Fig. S1).

To compare the continuous and pulsed models, we calculated the number of individuals removed under the continuous model and divided it by the number of pulses to compare equivalent removal efforts. At the end of the simulation, we recorded the average of the final 40 timesteps to account for variation around the equilibrium population size. For pulsed simulations where eradication occurred, we also recorded the mean time until eradication. We performed 10,000 replicate simulations, and code and all simulation parameters are available in the Supplementary Methods.

## Electronic supplementary material

Supplementary Information

## References

[1] Ostfeld RS, Keesing F (2000). Pulsed resources and community dynamics of consumers in terrestrial ecosystems. Trends Ecol. Evol..

[2] Yang LH (2010). A meta-analysis of resource pulse–consumer interactions. Ecol. Monogr..

[3] McCauley DJ (2012). Assessing the effects of large mobile predators on ecosystem connectivity. Ecol. Appl..

[4] Rasher DB, Hoey AS, Hay ME (2017). Cascading predator effects in a Fijian coral reef ecosystem. Sci. Rep..

[5] Silliman BR (2013). Consumer fronts, global change, and runaway collapse in ecosystems. Annu. Rev. Ecol. Evol. Syst..

[6] Butler MJ (1989). Community Responses to Variable Predation: Field Studies With Sunfish and Freshwater Macroinvertebrates. Ecol. Monogr..

[7] Navarrete SA (1996). Variable Predation: Effects of Whelks on a Mid-Intertidal Successional Community. Ecol. Monogr..

[8] McCoy Michael W, Barfield M, Holt Robert D (2008). Predator shadows: complex life histories as generators of spatially patterned indirect interactions across ecosystems. Oikos.

[9] Mailleret L, Lemesle V (2009). A note on semi-discrete modelling in the life sciences. Philos. Trans. R. Soc. Lond. Math. Phys. Eng. Sci..

[10] Shulgin B, Stone L, Agur Z (1998). Pulse vaccination strategy in the SIR epidemic model. Bull. Math. Biol..

[11] Agur Z, Cojocaru L, Mazor G, Anderson RM, Danon YL (1993). Pulse mass measles vaccination across age cohorts. Proc. Natl. Acad. Sci..

[12] Braverman E, Mamdani R (2008). Continuous versus pulse harvesting for population models in constant and variable environment. J. Math. Biol..

[13] Schaefer MB (1957). Some Considerations of Population Dynamics and Economics in Relation to the Management of the Commercial Marine Fisheries. J. Fish. Res. Board Can..

[14] Colvin ME, Pierce CL, Stewart TW, Grummer SE (2012). Strategies to Control a Common Carp Population by Pulsed Commercial Harvest. North Am. J. Fish. Manag..

[15] Cruz F, Josh Donlan C, Campbell K, Carrion V (2005). Conservation action in the Galàpagos: feral pig (Sus scrofa) eradication from Santiago Island. Biol. Conserv..

[16] Smith JE (2004). Ecology of the Invasive Red Alga Gracilaria salicornia (Rhodophyta) on O’ahu, Hawai’i. Pac. Sci..

[17] Holt RD (2008). Theoretical Perspectives on Resource Pulses. Ecology.

[18] Wright AN (2013). Pulses of marine subsidies amplify reproductive potential of lizards by increasing individual growth rate. Oikos.

[19] Boucek RE, Rehage JS (2013). No free lunch: displaced marsh consumers regulate a prey subsidy to an estuarine consumer. Oikos.

[20] Cortés-Avizanda A, Jovani R, Carrete M, Donázar JA (2012). Resource unpredictability promotes species diversity and coexistence in an avian scavenger guild: a field experiment. Ecology.

[21] McCoy MW, Wheat SK, Warkentin KM, Vonesh JR (2015). Risk assessment based on indirect predation cues: revisiting fine-grained variation. Ecol. Evol..

[22] Kain MP, McCoy MW (2016). Anti-predator behavioral variation among Physa acuta in response to temporally fluctuating predation risk by Procambarus. Behav. Processes.

[23] Piovia-Scott J, Yang LH, Wright AN (2017). Temporal Variation in Trophic Cascades. Annu. Rev. Ecol. Evol. Syst..

[24] Holling CS (1959). The Components of Predation as Revealed by a Study of Small-Mammal Predation of the European Pine Sawfly. Can. Entomol..

[25] Holling CS (1959). Some Characteristics of Simple Types of Predation and Parasitism. Can. Entomol..

[26] Hastings A, Hall RJ, Taylor CM (2006). A simple approach to optimal control of invasive species. Theor. Popul. Biol..

[27] Zipkin EF, Kraft CE, Cooch EG, Sullivan PJ (2009). When can efforts to control nuisance and invasive species backfire?. Ecol. Appl. Publ. Ecol. Soc. Am..

[28] Scheibling RE, Gagnon P (2006). Competitive interactions between the invasive green alga Codium fragile ssp. tomentosoides and native canopy-forming seaweeds in Nova Scotia (Canada). Mar. Ecol. Prog. Ser..

[29] Van Schalkwyk H, Potgieter L, Hui C (2017). The development of a spatio-temporal model for water hyacinth biological control strategies. Math. Comput. For. Nat.-Resour. Sci..

