# Supplementary material for: Top-down pulses reduce prey population sizes and persistence
Source: Sci Rep. 2018 Jun 19;8:9346. doi: 10.1038/s41598-018-27661-1 (PMC6008325; doi:10.1038/s41598-018-27661-1)
Supplement: Supplementary file 1 — Supplementary Information [file 41598_2018_27661_MOESM1_ESM.docx]

**Supplementary Information**

**Top-down pulses reduce prey population sizes and persistence**

**Elizabeth A. Hamman and Michael W. McCoy**


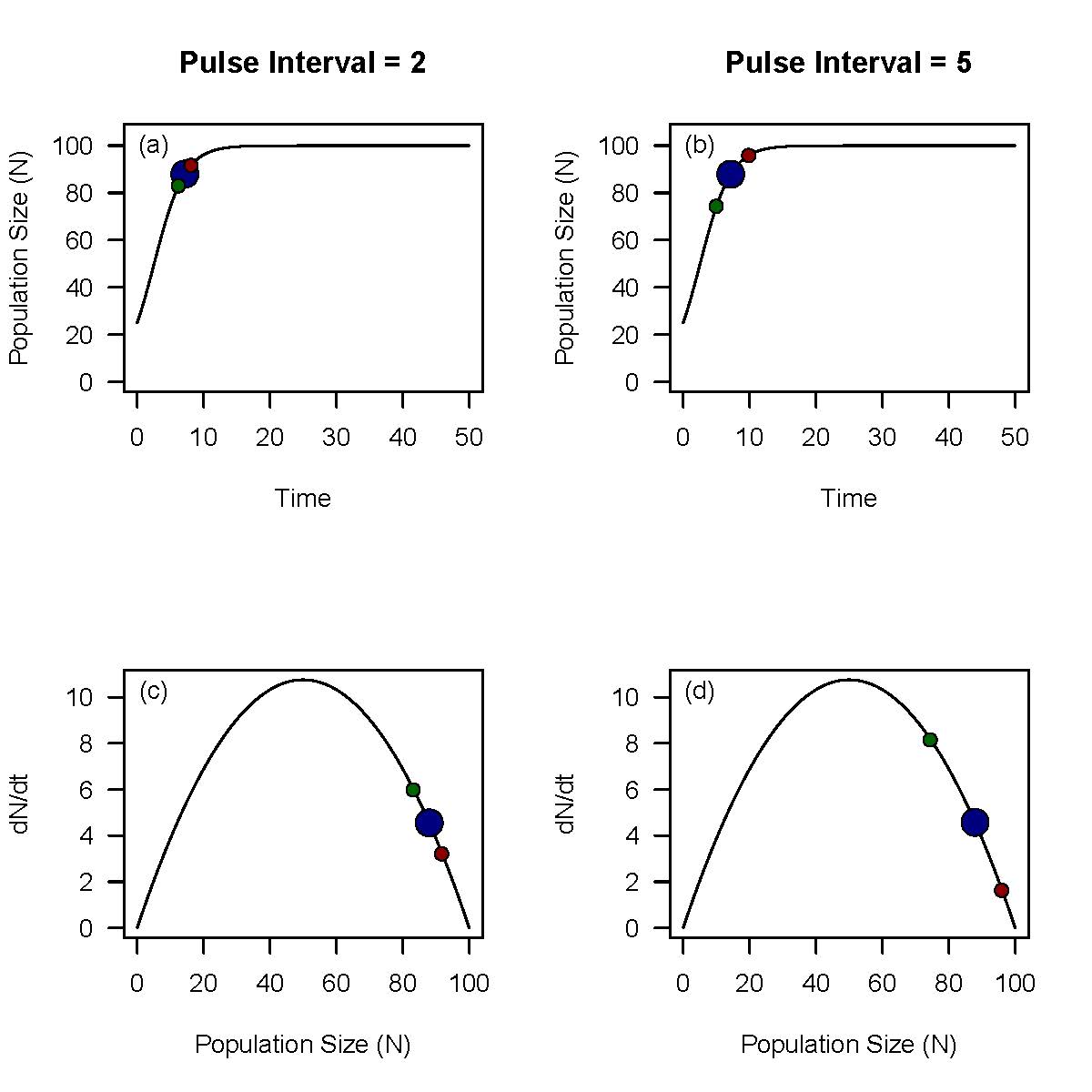


Supplementary Figure S1: Larger differences in population size reduction that occur with large, infrequent pulses are due to the nonlinearity in the population growth equation. In Panels a and c, the pulses are small and frequent, therefore the population size once the population has stabilized has a minimum size (green dot), maximum size (red dot), and equilbrium size (blue dot) very close in size, and with very little difference in dN/dt over the timeframe between pulses (Panel c). However, when pulses are larger and less frequent (Panels b and d), there is much more variation in population size (Panel b), and thus larger difference in dN/dt (Panel d) that result in lower population sizes.


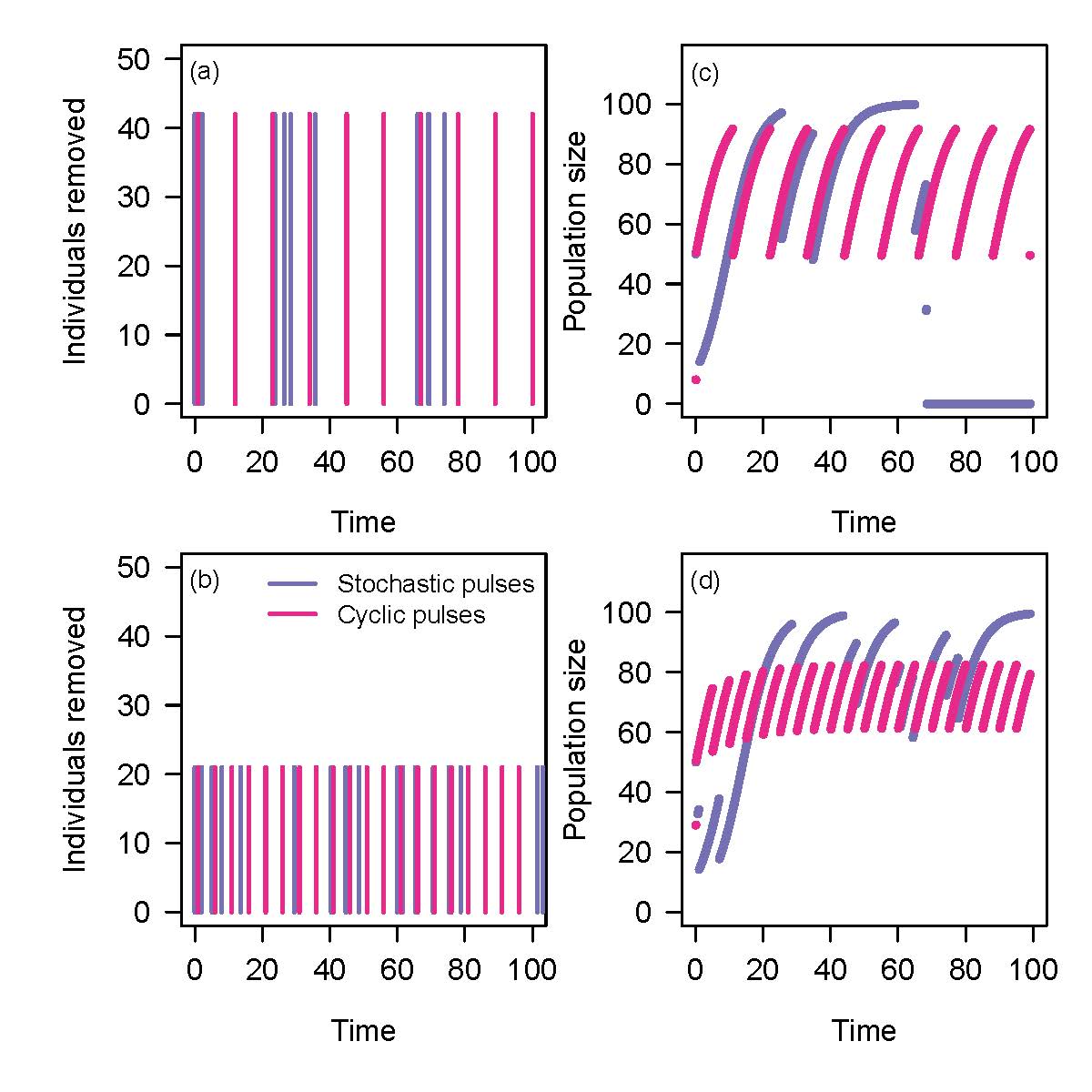


Supplementary Figure S2: Pulses spaced stochastically increase extinction probability due to the chance of sequential removal events decreasing the population quickly. Panels a and b show the timing of and size of stochastic (purple) and cyclic (pink) pulses. Corresponding population dynamics are shown in panels c and d. Pulses are rare (interval of 15) in panels a and c, and frequent (interval of 3.5) in panels b and d. In all panels, *H*=4.2 and *r*=0.22.

Supplementary Methods – R Code for Simulating Equations 1-3

# Overview

We simulated the solutions to equations 1-3 in R. For equations 1 and 2, we used the deSolve package. Here, we provide an example simulation with the following parameters

H <- 4.3 #removal rate
r <- 0.43 #intrinsic population growth rate
P <- 12.9 #interval between removal pulses
N0 <- 25 #initial population size
K <- 100 #carrying capacity

Time <- 100 #time simulation runs
timestep <- 0.1 #time interval for solutions

# No removals and continuous removals

Below, we define the functions for equation 1 (no removals), and equation 2 (continuous removals).

$$\frac{dN}{dt}=rN\left( 1-\frac{N}{K} \right)$$

No_Removals_ODE <- function (time, y, parms) {
 with(as.list(c(y, parms)), {
 dN <- r * N * (1 - N / K)
 list(dN)
 })
}

$$\frac{dN}{dt}=rN\left( 1-\frac{N}{K}-H \right)$$

Continuous_Removals_ODE <- function (time, y, parms) {
 with(as.list(c(y, parms)), {
 dN <- r * N * (1 - N / K) - H
 list(dN)
 })
}

For a given set of parameters, we selected a random amount of time for the simulation, generated the time vector, and used deSolve to solve the ODE.

library(deSolve)
rand_time <- round(runif(1,Time-0.1*Time, Time+0.1*Time)) # select a random
#length of the simulation (within 10% of set timesteps)
times <- seq(0, rand_time, timestep) # vector of timesteps for ode solver
y <- c(N = N0) # initial population size
parms_Logistic_Const <- c(r = r, H=H, K=K) # vector of parameters

No_Removals <- ode(y, times, No_Removals_ODE, parms_Logistic_Const)
Continuous_Removals <- ode(y, times, Continuous_Removals_ODE, parms_Logistic_Const)

# Pulsed removals

To simulate equation 3, $\frac{dN}{dt}=rN\left( 1-\frac{N}{K} \right)-\sum_{i=0}^{\infty} H\delta_{\gamma i}(t)$, we solved ode between removal pulses (logistic population growth), and used this combined with discrete pulses.

Logistic_growth <- function(N0,r,t,K){
 (K*N0*exp(r*t))/(K+N0*(exp(r*t)-1))
}

The time at which pulses occur depends on if pulses are cyclically, or regularly spaced, or if they are stochastically spaced. For stochastic pulses,

Stochastic_pulse_times <- function(num_timesteps,pulse_freq){

 # Select time between pulses (exponential RV with mean of cyclic pulse times)
 Actual_Pulse <- rexp(num_timesteps/pulse_freq,1/pulse_freq)

 Pulse_time <- c() # Empty vector for pulse times
 Pulse_time[1] <- 0 # First entry is 0

 # Create vector of pulse times with distances between pulses determined by Actual_Pulse
 for(i in 1:length(Actual_Pulse)){
 Pulse_time[i+1] <- Pulse_time[i]+Actual_Pulse[i]
 }

 Pulse_time <- round(Pulse_time,1) # round to the decimal points of "time increment"
}

We then simulated dynamics with the following function:

Pulse=function(num_timesteps, # time for simulations to run
 time_increment,# temporal resolution of simulations
 pulse_freq, #P, interval between pulses
 init_population, #N0
 stochastic, #0 for cyclic pulses, 1 for stochastic pulses
 pop_growth_rate, #r
 carrying_capacity, #K
 harvest_rate #H
 ){

 ## Step 1 - Determine the timing of the pulses using functions above ##

 if(stochastic==0){# Deterministic/regular pulses
 Pulse_time=seq(runif(1,min=1,max=pulse_freq),num_timesteps,by=pulse_freq) # Pulse times
 #are at regular intervals determined by pulse_freq with random timing for the first pulse
 Pulse_time=round(Pulse_time,decimalplaces(time_increment))
 }
 if(stochastic==1){ # Deterministic/regular pulses
 Pulse_time <- Stochastic_pulse_times(num_timesteps,pulse_freq)
 }


 ## Step 2 - Initialize Simulation
 Population <- c() # Empty vector for population sizes
 timesteps <- seq(1,num_timesteps,by=time_increment) # time increments for simulation

 # Determine harvest amount based on pulse frequency
 harvest_amount <- (harvest_rate*num_timesteps)/(length(Pulse_time))

 ## Step 3 - Complete first timestep
 # if it is a pulse, remove individuals from N0, otherwise leave at N0
 if(1 %in% Pulse_time==TRUE){
 Population[1] <- init_population-harvest_amount
 }
 if(1 %in% Pulse_time==FALSE){
 Population[1] <- init_population
 }

 ## Step 4 - Simulate population dynamics between pulses
 within_pulse_t <- 0
 for(t in 2:length(timesteps)){

 time <- timesteps[t] # match timestep to loop
 within_pulse_t <- within_pulse_t + time_increment # update "time" for within a pulse

 ## Pulsed Predation Events

 # if the timestep falls on a predetermined "pulse" event
 if(time %in% Pulse_time==TRUE&Population[t-1]>0){

 Population[t] <- Population[t-1] - harvest_amount

 init_population <- Population[t] # initial population is updated to include removals
 #(so continuous dynamics start with population right after a pulse)
 within_pulse_t <- 0 # update within pulse time tracker
 }

 if(time %in% Pulse_time==TRUE&Population[t-1]<=0){Population[t] <- 0}

 # Continuous time dynamics (if timestep is not a pulse)
 if(time %in% Pulse_time==FALSE){
 Population[t] <- Logistic_growth(init_population,pop_growth_rate,
 within_pulse_t,carrying_capacity)
 }

 if(Population[t] < 0){
 Population[t] <- 0 # if more individuals were removed than exist, N=0
 init_population <- 0
 }

 }
 return(rbind(timesteps-1,Population))
}
